# COVID-19 pandemic – an African perspective

**DOI:** 10.1080/22221751.2020.1775132

**Published:** 2020-06-15

**Authors:** Shabir Ahmad Lone, Aijaz Ahmad

**Affiliations:** a Clinical Microbiology and Infectious Diseases, School of Pathology, Health Sciences, University of the Witwatersrand, Johannesburg, South Africa; b Infection Control, Charlotte Maxeke Johannesburg Academic Hospital, National Health Laboratory Service, Johannesburg, South Africa

**Keywords:** SARS-CoV-2, COVID-19, coronavirus, Africa, Global warming, Epidemiology

## Abstract

The recently emerged novel coronavirus, “severe acute respiratory syndrome coronavirus-2 (SARS-CoV-2)”, caused a highly contagious disease called coronavirus disease 2019 (COVID-19). The virus was first reported from Wuhan city in China in December, 2019, which in less than three months spread throughout the globe and was declared a global pandemic by the World Health Organization (WHO) on 11th of March, 2020. So far, the ongoing pandemic severely damaged the world's most developed countries and is becoming a major threat for low- and middle-income countries. The poorest continent, Africa with the most vulnerable populations to infectious diseases, is predicted to be significantly affected by the ongoing COVID-19 outbreak. Therefore, in this review we collected and summarized the currently available literature on the epidemiology, etiology, vulnerability, preparedness and economic impact of COVID-19 in Africa, which could be useful and provide necessary information on ongoing COVID-19 pandemics in the continent. We also briefly summarized the concomitance of the COVID-19 pandemic and global warming.

## Introduction

There are hundreds of viruses that belong to the coronavirus family. However, only six (229E, NL63, OC43, HKU1, SARS-CoV and MERS-CoV) have been reported to cause mild to severe respiratory tract infections in humans [1]. Among them are severe acute respiratory syndrome coronavirus (SARS-CoV) reported in November 2002 and middle east respiratory syndrome coronavirus (MERS-CoV) reported in September 2012, which emerged in human population from animal reservoirs and caused severe respiratory illness with high mortality rates [2,3]. Once again, a novel severe acute respiratory syndrome coronavirus-2 (SARS-CoV-2) has emerged, and caused an infectious disease called coronavirus disease 2019 (COVID-19) [4]. The virus was first identified and reported from Wuhan city of China in December, 2019 [5]. The SARS-CoV-2 is highly contagious, spread globally in a short period of time, and was declared a global pandemic by the World Health Organization on March 11, 2020 [6]. As of 18th April, 2020, 10:00am CEST; WHO reported more than 2.1 million confirmed cases of COVID-19, including 142,229 deaths in 213 countries, areas or territories [7]. The most-affected countries with more than 30,000 confirmed cases of SARS-CoV-2 are the United States of America, Spain, Italy, Germany, France, the United Kingdom, China, Iran, Turkey, Belgium, the Russian Federation, Canada and Brazil [7]. However, the number of cases continues to rise throughout the globe and became a serious menace to public health.

COVID-19 is majorly affecting many countries all over the world, whereas Africa is the last continent to be hit by the pandemic. However, Africa is expected to be the most vulnerable continent where COVID-19 spreading will have a major impact [8]. The continent confirmed its first case of COVID-19 in Egypt on 14th of February, 2020, and from sub-Saharan Africa the first case was reported in Nigeria on 27th of February, in an Italian patient who flew to Nigeria from Italy on 25th of February, 2020 [9,10].

As of 18th April 2020, 10:00 am CEST; Africa CDC reported, 19,895 confirmed cases, including 1,017 deaths and 4,642 recoveries, from 52 African countries, while two countries (Comoros and Lesotho) were still virus-free [11]. Interestingly, most of the identified cases of COVID-19 in Africa have been imported from Europe and the United States, rather than from the original COVID-19 epicentre China [12].

The continent's weak health care system and a large immunocompromised population owing to high prevalence of malnutrition, anemia, malaria, HIV/AIDs, tuberculosis and poor economic discipline, make it distinct from the other continents that have experienced COVID-19 to date [13]. Experts also anticipated that under these circumstances the pandemic in Africa could be challenging to control, and the consequences could be dismal [13]. On the other hand, there is no drug/vaccine currently available to treat COVID-19; therefore, implementation of precautionary measures to contain the spread of this virus is being practiced throughout the globe; which includes social distancing, isolation and quarantine, community containment, national lockdowns, and travel restrictions. So far, these measures are helping to control and reduced the spread of COVID-19; but subsequently hit the global economy and thereby pushing the nations towards recession [14,15]. African economies were already struggling when COVID-19 hit the continent; which could further amplify the economic crisis. A unique COVID-19 response needs to be developed for Africa, where all these issues which make the continent more vulnerable and different to the rest of the world, will be taken into consideration.

## Aim of this review

Although there are numerous excellent research publications addressing this new menace, however this review aims to provide a comprehensive update of ongoing COVID-19 pandemics in Africa and highlighted the main topics which include; etiology, epidemiology, vulnerability, preparedness and economic impact of COVID-19 in African continent. It is therefore hoped that this work could be useful in addressing the continent's challenges related to the outbreak and will become the benchmark reference for future studies.

## Study selection

We searched and reviewed published work on COVID-19 from 1st January, 2020 to 18th of April, 2020. PubMed, ScienceDirect and Scopus were searched for research articles written on COVID-19 in English using the search words SARS-CoV-2, COVID-19 and coronavirus. The search was done in duplicate by two different individuals for the reproducibility with exclusion criteria for non-English articles and non-COVID-19 papers. After deduplication and exclusion, 54 out of 71 published articles as on 18/04/2020 were included. Reports and updates from the World Health Organization (WHO), the World Health Organization African Region, Centers for Disease Control and Prevention (CDC), Africa Centers for Disease Control and Prevention, and from other authentic sources were added. The results were grouped and systematically presented in this review.

## Etiology of COVID-19

The uncertainty about the SARS-CoV-2 origin is still an important aspect of this pandemic, and needs much attention to stop like ones in future. Initially there were reports that suggested the virus may have originated from bats, which are already known as a natural reservoir for various CoVs, including SARS-CoV-like and MERS-CoV-like viruses [16–20]. Upon phylogenetic analysis it has now been shown that there is a 96.2% sequence identity of SARS-CoV-2 with a coronavirus isolated from a bat (BetaCoV/RaTG13/2013) [21]. Furthermore, the genetic sequence of SARS-CoV-2 also shares >80% and 50% sequence identity to SARS-CoV and MERS-CoV respectively [22,23]. Thus, these findings indicate that the COVID-19 belongs to genus β-CoVs that infects humans, bats and other wild animals [24]. Other reports also suggested the possibility of virus transmission from bats to humans through unknown intermediate hosts [25]. Forster and colleagues recently analysed 160 complete human SARS-Cov-2 genomes by using a phylogenetic network analysis, and came up with some interesting findings [26]. The results revealed three distinct “variants” of COVID-19, consisting of clusters of closely related lineages, which they label “A”, “B” and “C”. They found that type “A” is closest to the one discovered in bats and is the ancestor to all other variants. Most cases of the COVID-19 in the United States and Australia were type “A”. Type “B”, only separated by two mutations from the ancestor “A”, was prevalent in China and other East Asian countries. Type “C”, predominantly found in patients in European countries, showed very little linkage with Type “B” [26]. So far, the genomic data is not sufficient and clear to prove the true origin and transmission source of SARS-CoV-2. Studies even seem to contradict previous hypotheses, which considered Wuhan, the city in China, as the origin of COVID-19. However, more sequencing is needed, using samples from other wild animals such as turtles, pangolins and snakes, which may play a possible role as intermediate hosts to solve this puzzle and confirm the origin of SARS-CoV-2. Compared to the global 7,700 genome sequences of SARS-CoV-2, the African continent has just pooled 90 genome sequences for this virus [27]. Additionally these sequences are coming from only 5 out of 51 infected countries, leaving a data dark spot in the continent [27]. Considering the mutations of the virus and the importance of this data for vaccine developments, African countries need to contribute more to the global genomic data pool; otherwise Africans will be facing the same problem as with the Rotavirus vaccine. The vaccine was developed based on rotavirus strains predominantly found in Europe and North America for use against rotavirus infections. However, the vaccine exhibited efficacy variation, seems more effective in Europe and North America but less effective in Africa and is believed due to the circulation of different strain in the continent [27].

## Epidemiology of COVID-19 in Africa

Starting from Wuhan City, Hubei Province of China (Original epicenter of COVID-19) and spreading around the globe in less than 3 months, the COVID-19 pandemic is considered the one among the biggest pandemics to humans [5,28]. As the pandemic is still ongoing, the number of countries involved, confirmed cases and mortality rates are changing every day. As the virus enters different countries at different time points, these countries are at different stages of the outbreak. With this complicity, true epidemiology is only possible at the end of this pandemic. As of 18th April, 2020, the novel SARS-CoV-2 has emerged in all seven continents and affects 213 countries and territories with 2,121,675 confirmed cases, and a mortality rate of 6.7% [7]. To date, the top three most-affected countries with COVID-19 include the United States of America (confirmed cases at 665,330 and 4.6% mortality), Spain (confirmed cases 182,816 and 10.5% mortality), and Italy (confirmed cases 168,941 and 13.1% mortality)[7].

With the currently available data, we attempted to monitor and track the epidemics of SARS-CoV-2 in the African continent. The African continent is the last one and least to be affected by COVID-19 pandemic to date [29]. As of 18th April, 2020, Africa reported 19,895 confirmed cases from 52 countries with a mortality rate of 5.1% [11]. First seen in Egypt on 14th of February 2020, the virus has now been detected in almost all the countries of Africa except Lesotho and Comoros [11]. Chronologically, Egypt was followed by Algeria, with its first case reported on 25th February, followed by Nigeria on 27th of February [30]. Apart from these three countries, the first cases in other African countries were only detected in March (Table 1) [11]. The most-affected countries so far are South Africa (confirmed cases = 2783, mortality = 1.8%), Egypt (confirmed cases=2844, mortality = 7.2%), Morocco (confirmed cases = 2564, mortality = 5.3%), Algeria (confirmed cases = 2418, mortality = 15.0%) and Cameroon (confirmed cases = 1016, mortality = 2.1%) [11]. However, due to inadequate testing capacity for COVID-19 the true number of cases may remain undetected, which makes it challenging to predict or conclude the true epidemiology of COVID-19 in the continent. Certainly, several major factors, such as late arrival of the pandemic, weak diagnostics including inadequate COVID-19 testing, lack of essential medical supplies and a large susceptible population will significantly affect and change the epidemiology of COVID-19 in the continent [13,31]. The epidemiological data of COVID-19 from the African continent are summarized in Table 1, which include: number of affected countries with confirmed cases, deaths and recovery cases [11].

**Table 1.** Epidemiology of COVID-19 cases in Africa as of 18th of April, 2020.

Country	Confirmed cases	Deaths	Recoveries	First case/s
Algeria	2,418	364	846	25th Feb, 2020
Angola	19	2	6	21st Mar, 2020
Benin	35	1	18	16th Mar, 2020
Botswana	15	1	0	30th Mar, 2020
Burkina Faso	557	35	294	9th Mar, 2020
Burundi	6	1	4	31st Mar, 2020
Cameroon	1,016	21	168	6th Mar, 2020
Cape Verde	56	1	1	20th Mar, 2020
Central African Republic	12	0	5	14th Mar, 2020
Chad	33	0	8	19th Mar, 2020
Comoros	0	0	0	NA
Congo-Brazzaville	143	6	11	10th Mar, 2020
DR Congo	307	25	26	10th Mar, 2020
Djibouti	732	2	76	18th Mar, 2020
Egypt	2,844	205	646	14th Feb, 2020
Equatorial Guinea	79	0	3	14th Mar, 2020
Eritrea	35	0	0	20th Mar, 2020
Eswatini	19	1	8	14th Mar, 2020
Ethiopia	96	3	15	13th Mar, 2020
Gabon	108	1	7	12th Mar, 2020
Gambia	9	1	2	17th Mar, 2020
Ghana	641	8	83	12th Mar, 2020
Guinea	477	3	59	13th Mar, 2020
Guinea-Bissau	50	0	0	25th Mar, 2020
Ivory Coast	742	6	220	11th Mar, 2020
Kenya	246	11	53	12th Mar, 2020
Lesotho	0	0	0	NA
Liberia	76	7	7	16th Mar, 2020
Libya	49	1	11	24th Mar, 2020
Madagascar	117	0	33	20th Mar, 2020
Malawi	17	2	0	2nd Apr, 2020
Mali	190	13	34	25th Mar, 2020
Mauritania	7	1	2	13th Mar, 2020
Mauritius	324	9	108	19th Mar, 2020
Morocco	2,564	135	281	2nd Mar, 2020
Mozambique	34	0	2	22nd Mar, 2020
Namibia	16	0	4	14th Mar, 2020
Niger	627	18	110	19th Mar, 2020
Nigeria	493	17	159	27th Feb, 2020
Rwanda	143	0	65	14th Mar, 2020
Sao Tome and Principe	4	0	0	6th Apr, 2020
Senegal	342	3	198	2nd Mar, 2020
Seychelles	11	0	5	14th Mar, 2020
Sierra Leone	26	0	0	31st Mar, 2020
Somalia	116	5	2	16th Mar, 2020
South Africa	2,783	50	903	5th Mar, 2020
South Sudan	4	0	0	5th Apr, 2020
Sudan	32	6	5	13th Mar, 2020
Tanzania	147	5	11	16th Mar, 2020
Togo	83	5	48	6th Mar, 2020
Tunisia	864	37	43	2nd Mar, 2020
Uganda	55	0	20	20th Mar, 2020
Zambia	52	2	30	18th Mar, 2020
Zimbabwe	24	3	2	15th Mar, 2020

## Vulnerability and preparedness for COVID-19 in Africa

The COVID-19 pandemic is a wake-up call for Africa: the high burden of infectious diseases, weak health systems, poverty and the arrival of the winter “flu” season in Southern Africa, are some major factors which particularly make the continent one of the most vulnerable to this current pandemic. According to the Infectious Disease Vulnerability Index (IDVI) 2016, out of 25 countries most vulnerable to infectious diseases, 22 are in the African region [9]. The WHO Africa estimated that there are 26 million people infected with HIV, 2.5 million with tuberculosis, 71 million with hepatitis B or C and 213 million with malaria in the African region [32–35]. Moreover, the double burden of noncommunicable diseases (NCDs) such as cardiovascular diseases, cancers, chronic respiratory diseases and diabetes are also immensely significant in Africa, and all these conditions compromise the body's immunity [36,37]. Therefore, it could be reasonably hypothesized that the majority of the African population, due to their immunocompromised conditions, will be at high risk for COVID-19.

A country's healthcare capacity plays a vital role in COVID-19 management and control [38]. In comparison to the developed nations such as USA, the UK and China, which have advanced health care systems but are still struggling to cope with the current pandemic, the majority of African countries have a weaker healthcare sector [38–40]. The limited testing capacity, shortage of trained staff required for diagnostics and intensive care units (ICU), inadequate ventilators and ICU facilities (needed in severe cases of COVID-19), lack of personal protective equipment (PPE) for healthcare workers and scarcity of funds for the health sector, are some of the continent's core healthcare related issues, which make it more susceptible to the COVID-19 pandemic [38–41]. The other misfortune for Southern Africa is the arrival of winter, as all respiratory viruses spread more effectively in the winter; thus it is anticipated that the intensity of COVID-19 will increase in the coming winter months between May and September 2020 [42–44]. On the other hand, this shift in the seasons may become fortunate for northern hemisphere countries, where summer is coming and will likely decrease the transmission of SARS-CoV-2 [42].

As we are witnessing how the ongoing pandemic is hampering the world's most developed countries which have advanced healthcare, a low disease burden and established economies; it will be interesting to see the impact of COVID-19 on low- and middle-income countries. Unfortunately most of the African countries fall into this category, therefore it may be challenging for them to cope with the COVID-19 pandemic. Experts have already predicted that the growth of the African continent will be significantly impacted by the ongoing COVID-19 outbreak [45–47]. However, the magnitude of the impact will depend on the management and control of COVID-19 within the respective countries. Recently, a modelling study based on the State Party Self-Assessment Annual Reporting (SPAR), Index and Infectious Disease Vulnerability Index (IDVI), measured the preparedness and vulnerability of African countries against COVID-19 importations from China [48]. Both indicators (SPAR and IDVI) ranged from 0 to 100, measure increasing capacity and decreasing vulnerability. The study reported that Egypt, Algeria and South Africa had the highest importation risk from China, with the SPAR scores of 87, 76, and 62 respectively, and have moderate to high capacity to respond to outbreaks, with IDVI scores of 53, 49, and 69 respectively [48]. Countries such as Nigeria and Ethiopia have moderate importation risk, with SPAR scores of 51 and 67 respectively, but high vulnerability with IDVI scores of 27 and 38 respectively. Sudan, Angola, Tanzania, Ghana, and Kenya also have similar moderate importation risk with variable levels of capacity (ranging from 34 to 75), and an overall low IDVI (<46), reflecting high vulnerability [48]. On a positive note, the demography of the African continent seems to be an advantage when compared to other COVID-19 affected regions. The median age in Africa is less than 20, that makes the continent the youngest in the world [38]. Only 4% of Africa's population is older than 65, which are low as compared to 37% in Eastern and South-Eastern Asia and 29% in Europe and Northern America [49]. Current data suggests that COVID-19 affects older people severely, with higher mortality than the younger population, which showed only milder symptoms [38]. In addition, Africa is the last continent to be hit by COVID-19, and therefore gets some extra time with additional information for preparations to face the pandemic. Africa also had lessons to be learnt from other countries and from the previous outbreaks, to act urgently on specific weaknesses and implement strict measures of detection, prevention, and control to enhance preparedness for COVID-19 pandemic. Recently, several strategic measures, which include complete lockdowns, travel bans, closing of schools, companies, and offices, ban on large gatherings (including religious, sports, social and other events), systematic quarantines, increased testing capacity and strict infection control measures, are being implemented throughout the African continent to control the spread of COVID-19 [50]. Furthermore, on 5th February 2020, the African task force for coronavirus (AFCOR) was established by Africa CDC in collaboration with the African Union Commission (AUC) and the WHO, to step up the preparedness measures for COVID-19 closure [51]. The AFCOR aims to focus on six work streams: laboratory diagnosis and subtyping, surveillance including screening at points of entry and cross-border activities, infection prevention and control in healthcare facilities, clinical management of people with severe COVID-19, risk communication, supply-chain management and stockpiles. The measure breakthrough for preparedness is in terms of laboratories testing for SARS-CoV-2 in the African continent. On 6th of March 2020, Africa CDC reported that 43 African countries are now able to test for COVID-19, while as at February 2020, only two countries (Senegal and South Africa) were capable of diagnosing the virus [38]. The big support from the World Bank will also assist developing countries for COVID-19 preparedness and response. In Africa, immediate support of $82 million for Ethiopia and $47 million for the Democratic Republic of Congo have been approved [52]. These figures for other African countries are yet to be confirmed; however, there are big supports from several agencies, Non-Governmental Organizations (NGOs), philanthropists, funding agencies and banks to all the African nations, in order for them to be prepared for the ongoing COVID-19 pandemics. Nevertheless, it would not be ideal to suggest that this support will be enough to prepare these countries for this pandemic.

As discussed above, owing to several reasons, Africa is found to be at high risk for COVID-19 pandemic, with relatively low capacity to manage the health emergency. Therefore, urgent attention, support and action are required to fight and control the further spread of the ongoing pandemic.

## Economic impact of COVID-19 in Africa

The initial phase of the COVID-19 pandemic was all about clinical and epidemiological aspects however, the shift is now changing towards the global economy. The focus of effect of COVID-19 pandemics needs to shift to the developing nations, and particularly to African countries which rely mostly on developed countries. Economists had estimated Africa's growth in 2020 at 3.9%, which can now drop to 0.4% (in the best case) to −3.9% (in the severely hit case) [45,46]. Experts also believe that growth in Sub-Saharan Africa may fall to between −2 and −5% in comparison to 2.4% in 2019, with a risk of the first recession in the last 25 years [47]. The major factors which may affect the African economy related to COVID-19 are:

1. Reduction of importation of Chinese goods to the level that it inflates the African markets. This will have a further impact on the small scale traders of developing markets, and will increase the prices of local commodities.
2. Decreasing oil consumption due to travel bans, border closures, social distancing and lock downs lowering down the demand for oil. The budget of some of the African oil-producing countries such as Nigeria, Angola, Algeria, Ghana and others, is dependent upon crude oil pricing, which has been badly hit by this pandemic, thereby impacting the GDP of these countries [41,53]. This could however have a positive impact on oil-importing countries.
3. African mining industry: The mining sector is China's top most interest for investing in Africa than any other big economy. Travel restrictions, shutdowns and port closures have resulted in decreasing demand for steel, iron ore, lithium, and cobalt [41]. Alone in South Africa, the mining industry employs around 420,000 people and thousands of them are working underground which suggests that the mining work environment is more exposed to pandemic and can become a catalyst for spreading the COVID-19 [54]. As such, the African mining sector faces an unavoidable hit from the ongoing COVID-19 pandemic, even though there is still much uncertainty as to how much and for how long the sector will be impacted.
4. Reduction of tourism: The major economic sector of many African countries such as South Africa, Ethiopia, Kenya and Tanzania is tourism, which is negatively affected due to COVID-19, thereby affecting the economies of these countries [41,53].
5. Withdrawal of investors: Developing markets already taste the bitterness where investors have already fled, with the largest capital flow ever recorded [53]. Foreign direct investments have already been declined due to delays or cancellation of several revenue boosting projects. Also, the flow of aid and other assistance projects have been stopped, as the donor countries are themselves struggling with the same pandemic situation.
6. The shift of budgets from other sectors to the health sector is a timely need, and this will cause a further decline in the economic growth of these countries.
7. The lower revenue will in turn reduce the tax rates; which will badly impact on the fiscal revenues of poor countries in Africa [53].

All these factors will put governments under extreme pressure in preparing for the post-crisis of the COVID-19 pandemic. Experts are calculating around 20 million job losses, which will further increase the unemployment rates of African countries [53]. Increase in unemployment could possibly lead to social unrest and increasing crime rates in the countries with a history of sectarian violence.

## Concomitance of COVID-19 pandemic and global warming

Although there is no scientific evidence so far to show any direct link between global warming and the COVID-19 pandemic, scientists are giving opinions that these two run parallel to each other. Being a zoonotic virus (bat species suspected to be SARS-CoV-2 virus reservoir), there are several reasons to connect the COVID-19 pandemic to climate change. Global warming along with other associated factors such as habitat destruction, human encroachment, modernization of farming, etc., have been reported to drive the emergence of zoonotic diseases [55]. In a study by Naicker, a link between the climate changes with the zoonotic diseases outbreaks such as West Nile fever, Chikungunya fever and Lyme disease has been well described [56]. Climate changes have been reported to impact pathogens selection and resistance, hosts ecology and immunity, as well as vectors ecological niches and capacity; with more potential influence on vector-borne and zoonotic diseases [57]. In addition, glaciers which are hidden sources of numerous pathogens especially viruses, are melting due to globally increasing temperatures and the resident pathogens are therefore getting a wakeup call [58]. Melting glaciers are liberating these pathogens, including those which are new to science [59]. Climate change is also associated with deforestation and encroachment into animal habitats, which forced several wild species to migrate and thereby putting these species in close contact with humans and other animals [60]. Unplanned migrations also increases the stress levels in these species, leading to immunocompromised conditions, which subsequently increase the tendency of increased risk of infections and increased viral replication [61]. Despite all these reasons, there is no concrete evidence to prove the claims of linking the two to each other. However as COVID-19 is still unfolding, the underlying links between global warming and the spread of this virus may be unveiled.

As of the current date, China is the world's largest CO_2_ emitter followed by the United States the European Union, the Indian sub-continent and the Russian Federation [62,63]. On the other hand, the African continent is the lowest contributor of greenhouse gas emissions, with very low per capita CO_2_ emissions compared with other continents [62]. According to the World Resources Institute, Africa has been responsible for less than 0.01% of all emissions [62]. Despite being the lowest contributor, the African continent is the worst sufferer of any climate change related adversity, ranging from economic growth and sustainable development to infectious diseases [64]. However, in the case of the COVID-19 pandemic the trend is different; where African continent is the least affected so far. It is far too early to reach a conclusion, as experts are warning that the rise of infection peak in Africa is yet to materialize. The origination and spread of SARS-CoV-2 virus in the biggest CO_2_ emitting countries is higher than in African countries [7,29]; which could be mere coincidence. Within the African continent, a similar trend was observed with South Africa being the major contributor in total greenhouse gas emissions and higher number of confirmed COVID-19 cases [7,62]. All the figures, support being optimistic at this stage about developing some models which can identify the links between climate change and current and future pandemics. It is also believed that this pandemic will offer an opportunity to understand the further consequences of climate change and related pandemics.

## Conclusion

As of now, COVID-19 continues to spread globally, with increasing morbidity and mortality, with some control in the African continent compared to the other parts of the world. The swift actions against this pandemics imposed by the governments have been effective so far. However, as the majority African population is living from hand to mouth, these measures cannot sustain for long. Some countries including South Africa and Ghana have already started lifting or relaxing these restrictions due to the high impact on their economies. Therefore besides these restrictions other mitigation strategies, to improve economies and to provide basic benefits to public, need to be implemented by the governments. Based on past experiences, there is a scope of suppressing transmission of COVID-19, provided governments and the public will change their behaviour towards this virus as they did previously for Ebola, HIV, Polio and other outbreaks. However, it comes as no surprise that Africans can't confront this alone, and therefore global support in any form can assist Africa to step ahead of this pandemic.
